# Impact of Modifying Lazertinib Doses on Effectiveness and Safety in Patients With EGFR‐Positive Advanced Lung Cancer: A Multicenter, Prospective Observational Cohort Study

**DOI:** 10.1111/1759-7714.70083

**Published:** 2025-05-21

**Authors:** Mi‐Hyun Kim, Min Ki Lee, Ji Eun Park, Sun Hyo Park, Tae Won Jang, Chi Young Jung, Insu Kim, Seong Hoon Yoon, June Hong Ahn, Hyun‐Kyung Lee, Jin Han Park, Sun Ha Choi, Jung Seop Eom

**Affiliations:** ^1^ Department of Internal Medicine Pusan National University School of Medicine Busan Republic of Korea; ^2^ Department of Internal Medicine, School of Medicine Kyungpook National University Daegu Republic of Korea; ^3^ Division of Pulmonology, Respiratory Center, Keimyung University Dongsan Hospital Keimyung University School of Medicine Daegu Republic of Korea; ^4^ Department of Internal Medicine Kosin University Medical College Busan Republic of Korea; ^5^ Department of Internal Medicine Catholic University of Daegu School of Medicine Daegu Republic of Korea; ^6^ Department of Internal Medicine, College of Medicine Dong‐A University Busan Republic of Korea; ^7^ Department of Internal Medicine Pusan National University Yangsan Hospital Yangsan Republic of Korea; ^8^ Department of Internal Medicine, College of Medicine Yeungnam University Daegu Republic of Korea; ^9^ Division of Pulmonary, Allergy, and Critical Care Medicine, Department of Internal Medicine, Inje University Busan Paik Hospital Inje University College of Medicine Busan Republic of Korea; ^10^ Division of Pulmonology and Critical Care Medicine, Department of Internal Medicine, Haeundae Paik Hospital Inje University College of Medicine Busan Republic of Korea; ^11^ Department of Internal Medicine, School of Medicine Kyungpook National University Chilgok Hospital Daegu Republic of Korea

**Keywords:** dose modification, epidermal growth factor receptor, lazertinib, treatment outcome

## Abstract

**Introduction:**

The clinical application of lazertinib, a third‐generation epidermal growth factor receptor (EGFR) tyrosine kinase inhibitor, has extended to the treatment of EGFR‐mutant non‐small‐cell lung cancer (NSCLC); however, the effects of its dose modification on its efficacy and safety have not yet been adequately established.

**Methods:**

This prospective, multicenter, observational cohort study aims to evaluate the clinical implications of adjusting the lazertinib dose. Patients will be categorized into two groups based on the lazertinib dose administered during the initial 12 weeks of treatment in routine clinical practice: 160 and 240 mg groups. The primary endpoints are progression‐free survival in the 160 mg group and identifying risk factors associated with dose modification during the 12‐week period.

**Discussion:**

The findings from the present study will provide real‐world insights into the clinical factors leading to lazertinib dose adjustments and deepen our understanding of the efficacy and safety of lazertinib in patients with NSCLC. Our research will contribute toward optimizing medical strategies for NSCLC treatment and aid clinicians in making accurate clinical decisions regarding dose modifications in routine practice.

## Graphical abstract

- Lazertinib dose optimization protocol for non‐small‐cell lung cancer (NSCLC).
- This prospective study evaluates the efficacy and safety of Lazertinib at various doses in patients with EGFR‐mutant non‐small‐cell lung cancer (NSCLC) to identify optimal dosing strategies and associated risk factors for dose modifications.

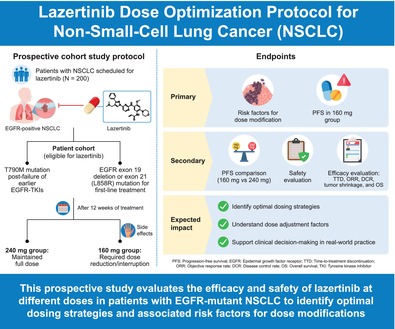

## 1 Introduction

Cancer remains the leading cause of mortality in South Korea and worldwide. According to a report on the 2021 cancer statistics for Korea, lung cancer ranks as the leading cause of cancer‐related mortality in Korea, followed by liver, colorectal, gastric, and pancreatic cancers [1]. However, the development of new and effective treatments, such as targeted therapies, has substantially increased the survival of patients with non‐small‐cell lung cancer (NSCLC) harboring mutations sensitive to these targeted therapies [2].

Among these mutations, those in the epidermal growth factor receptor (EGFR) gene are highly prevalent in East Asian populations, with a reported incidence of approximately 30%–50% [3]. Based on the findings of the FLAURA trial, currently, osimertinib, a third‐generation tyrosine kinase inhibitor (TKI), remains the standard‐of‐care first‐line treatment recommended for patients harboring EGFR‐sensitizing mutations (exon 19 deletion or exon 21 L858R substitution) [4, 5]. Moreover, other third‐generation TKIs, which have been demonstrated to be superior to first‐generation TKIs, have been approved for use in different countries. Lazertinib, a third‐generation TKI, is an irreversible, highly selective EGFR‐TKI that spares wild‐type EGFR while effectively targeting active EGFR mutations, including exon 19 deletion, L858R, and T790M. Lazertinib has been established as an effective treatment for EGFR‐mutant NSCLC, either as monotherapy or in combination with other agents, in both first‐ and subsequent‐line settings, as demonstrated in the LASER301, MARIPOSA, and MARIPOSA‐2 trials [6, 7, 8]. However, dose reductions are often required in some patients due to adverse effects (AEs), such as paresthesia. The efficacy and safety of lazertinib administered at a reduced dose of 160 mg have not yet been comprehensively assessed in real‐world settings.

Accordingly, in the present study, we aim to identify the risk factors associated with dose reductions in patients with NSCLC receiving lazertinib in routine clinical practice. Additionally, we seek to evaluate the efficacy and safety of lazertinib by comparing patients who are maintained on the initial 240 mg dose for 12 weeks after treatment initiation (240 mg group) with those who require a dose reduction to 160 mg (160 mg group).

## 2 Materials and Methods

### 2.1 Study Design

In the present study, which is based on a prospective, multicenter, and observational design, we aim to identify the risk factors associated with a reduction in lazertinib dosage in patients with NSCLC scheduled to receive treatment with this drug. Our study will include two patient cohorts: (1) those confirmed to harbor the EGFR T790M mutation after experiencing disease progression following treatment using first‐ or second‐generation EGFR‐TKIs and who are scheduled to receive lazertinib and (2) those confirmed to harbor the EGFR exon 19 deletion or exon 21 L858R substitution mutations and who are scheduled to receive lazertinib as a first‐line therapy.

**FIGURE 1:**
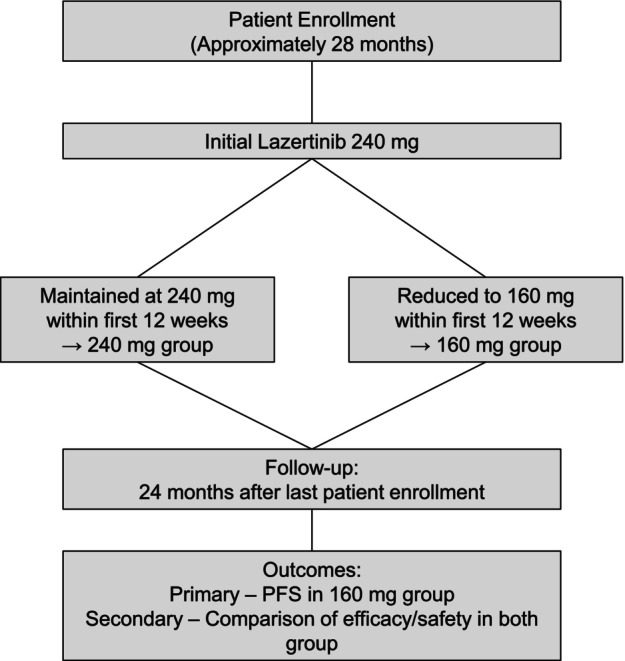
Flowchart of the study. The patients were grouped according to whether a dose reduction to 160 mg occurred within the initial 12 weeks of lazertinib treatment. PFS, progression‐free survival; AEs, adverse events.

The patients will not be randomized but instead will be classified into two groups based on their initial 12 weeks of treatment during routine clinical practice (Figure 1). Patients for whom the initial 240 mg dose of lazertinib is maintained during this period (without interruption or dose reduction) will be assigned to the 240 mg group; patients who require a temporary discontinuation of treatment and/or receive a reduced dose of 160 mg within the initial 12 weeks will be allocated to the 160 mg group. Although any subsequent dose escalation after the initial 12‐week window will be permitted at the discretion of the treating physician and recorded, this will not influence the initial group assignment. The efficacy and safety of lazertinib will be evaluated in both groups over a follow‐up period of up to 2 years from the date of last patient enrollment. Tumor assessments using CT imaging will be conducted every 8–12 weeks, according to routine clinical practice and at the discretion of the treating physician.

A total of 200 patients from 11 institutions in South Korea will be enrolled, with data collected through a review of electronic medical records.

### 2.2 Eligibility Criteria

Patients will be eligible for inclusion if they meet all of the following criteria: (a) provide written informed consent to participate in this study; (b) are aged 20 years or older at enrollment; (c) are diagnosed with locally advanced, metastatic, or recurrent NSCLC based on the 8th edition of the Cancer Staging Manual and Handbook of the American Joint Committee on Cancer (AJCC); and (d) are eligible for lazertinib treatment and meet at least one of the following conditions: (i) have confirmed presence of an EGFR T790M mutation in tumor tissues or plasma after the failure of first‐ or second‐generation EGFR‐TKI therapy; (ii) have confirmed presence of EGFR exon 19 deletion or exon 21 L858R substitution mutations and are scheduled to receive lazertinib as a first‐line treatment. Patients who are currently receiving or who have already completed lazertinib treatment and patients with an expected survival of less than 12 weeks will be excluded from this study.

### 2.3 Endpoints

Our primary objectives in this study are to assess the progression‐free survival (PFS) of patients in the 160 mg group within the initial 12 weeks after the initial administration of lazertinib and identify the potential risk factors associated with a reduction in dosage during this period. The PFS will be defined as the time from treatment initiation to radiological progression or death (whichever occurs first) based on CT imaging; the dates on which imaging is performed will be recorded to determine the progression events.

The secondary objective is to assess the efficacy and safety of lazertinib administered to patients in the two dose groups. Specifically, the PFS of patients in the 240 and 160 mg groups will be compared within the initial 12 weeks of treatment; further, the safety of lazertinib in the patients from both groups will be evaluated. Additional efficacy measures will include time‐to‐treatment discontinuation (TTD), objective response rate (ORR), disease control rate (DCR), tumor shrinkage, and overall survival (OS). We will also investigate the specific reasons for dose modifications in both groups and analyze the patient treatment profiles, including the treatment duration, dose adjustments, and reasons for treatment discontinuation.

### 2.4 Sample Size Estimation

The primary objective of this study is to estimate the PFS of the patients in the 160 mg group. On the basis of the findings of a previously conducted clinical trial, the median PFS in this population is assumed to be approximately 20.6 months [6]. To estimate the median PFS with a 95% confidence interval (CI) and a desired precision of ±4.5 months, we applied Greenwood's formula for survival analysis and assumed an exponential distribution of the survival time. Given these assumptions, approximately 15 events are required to achieve the desired level of precision. The study design includes uniform patient accrual over 28 months, with a 24‐month follow‐up period from the date at which the final patient was enrolled. This observational period is deemed sufficient to encompass the occurrence of events in the dose‐reduction group. Consequently, a minimum of 40 patients in the 160 mg group was deemed appropriate to meet the statistical requirements. Moreover, to ensure the inclusion of at least 40 patients in the dose‐reduction group, we estimated that the total sample size would need to be 200 patients; this is based on an expected dose reduction rate of approximately 20%–25% in real‐world clinical practice. Even at the lower bound of the anticipated rate (20%), enrolling 200 patients would yield 40 patients in the 160 mg group; thus, the target sample size for the analysis of this subgroup can be met.

### 2.5 Data Collection

Data will be collected by retrieving information from the electronic medical records of the patients.

Given the observational nature of this study, data collection will be limited to routine clinical assessments or tests conducted at the discretion of the investigators. Data regarding the following key variables will be collected:

– Baseline patient characteristics: date of birth, sex, age, ECOG performance status, comorbidities, concomitant medications, history of other cancers, and history of smoking;
– Pathological results: biopsy date, biopsy site, histological diagnosis, subtype of EGFR mutation, and other molecular profiles;
– Clinical stage at diagnosis and at the time of lazertinib administration based on the 8th edition of the AJCC;
– Treatment modality and clinical outcomes: date of imaging, history of lung cancer surgery, previous radiation therapy for lung cancer, previous systemic treatment for lung cancer, history of brain metastasis treatment, and history of bone metastasis treatment;
– Lazertinib dose modification;
– AEs according to the Common Terminology Criteria for Adverse Events version 5.0, including date of onset;
– Cases of treatment discontinuation due to AEs;
– Subject status: alive, dead, date of death, and date of last visit.

### 2.6 Statistical Analysis

Categorical variables will be summarized using frequencies and percentages (%) and analyzed using Pearson's chi‐square test or Fisher's exact test, as appropriate. Continuous variables will be summarized using the frequency, mean, and standard deviation (or median, minimum, and maximum) and analyzed using Student's *t*‐test or the Mann–Whitney *U* test, as necessary.

For survival or time‐to‐event variables, such as PFS and overall survival (OS), the Kaplan–Meier method will be employed to generate survival curves; the median survival and 95% CIs will be recorded. The groups will be compared using the log‐rank test; Cox regression modeling will be performed to identify the significant factors influencing dose reduction. For secondary comparisons between the 160 and 240 mg groups, exploratory multivariable analyzes (e.g., Cox proportional hazards models adjusted for relevant baseline characteristics) will be conducted to account for potential confounders. Missing data will not be imputed; all analyzes will be conducted based on as‐is collected data.

## 3 Discussion

In South Korea, lazertinib monotherapy was approved in 2021 for the treatment of patients with the EGFR T790M mutation in tumor tissues or plasma after unsuccessful treatment with either first‐ or second‐generation TKI agents [9]. Subsequently, on August 19th, 2024, the combination of lazertinib and amivantamab received approval from the US Food and Drug Administration as a first‐line treatment for locally advanced or metastatic NSCLC with EGFR exon 19 deletion or exon 21 L858R mutations [10]. These approvals underscore the expanding clinical utility of lazertinib. The use of the combination of amivantamab and lazertinib has led to increases in the PFS compared with that observed after osimertinib monotherapy; however, the frequency of AEs (grade 3 or higher) has increased by 75% (35% vs. 14%, respectively) [7]. This emphasizes the necessity to effectively control the toxicity of lazertinib to maintain antitumor efficacy. To date, however, neither the efficacy nor the safety of lazertinib at a reduced dose of 160 mg has been sufficiently established in real‐world settings. To the best of our knowledge, this is the first prospective cohort study to evaluate the efficacy and safety of lazertinib in the context of early dose modifications. We anticipate that the findings of the present study will enhance our understanding of the clinical factors influencing dose adjustments and provide real‐world insights into the efficacy and safety of lazertinib in patients with NSCLC.

## Author Contributions

**Mi‐Hyun Kim, Min Ki Lee, and Jung Seop Eom:** conceptualization. **Jung Seop Eom:** funding acquisition. **Ji Eun Park, Sun Hyo Park, Tae Won Jang, Chi Young Jung, Insu Kim, Seong Hoon Yoon, June Hong Ahn, Hyun‐Kyung Lee, Jin Han Park, and Sun Ha Choi:** investigation. **Mi‐Hyun Kim and Jung Seop Eom:** methodology. **Min Ki Lee and Jung Seop Eom:** project administration. **Mi‐Hyun Kim and Jung Seop Eom:** writing – original draft preparation.

## Ethics Statement

The study and protocol were approved by the Institutional Review Board of Pusan National University Hospital (No. 2212‐003‐132) and registered at ClinicalTrials.gov (NCT05716672).

## Consent

The study was conducted in accordance with the principles of the Declaration of Helsinki, and written informed consent was obtained from all study participants.

## Conflicts of Interest

The authors declare no conflicts of interest.

## Data Availability

The data used for this study is available from the corresponding author upon reasonable request.
